# Communications

**Published:** 1913-03

**Authors:** 


### Communications.

PRESIDENT'S OFFICE,
PARKE, DAVIS & CO.
DETROIT, MICH., February 6, 1913.

*Editor Texas Medical Journal, Austin, Texas.*

DEAR SIR: You have seen the attack which has been made upon our motives in marketing the Phylacogens, and upon the value of the products. That there is not a vestige of truth or warrant in the attack will be made very clear if you will glance at the "Laboratory Data," copy of which we are mailing to you under separate cover this day. These "Laboratory Data" were sent out by us in April, 1912, to physicians who were assisting us, in different parts of the United States, to ascertain, in the hospital and at the bedside, the true clinical value of the Phylacogens. No intelligent man can read these data and resist the conviction that they were prepared in a spirit of the greatest candor for the benefit of the physicians who were at work on the problem.

At the instance of Dr. Schafer we first began our independent investigation of his Phylacogens in January, 1911. It was not until March 1, 1912—fourteen months later—that we sold so much as a single dose. During that period over 40,000 packages were prepared and placed free of charge in the hands of our clinical coworkers. Since we began the marketing of the Phylacogens for rheumatism, gonorrheal rheumatism, erysipelas, and pneumonia, we have, of course, continued our experimental work in the laboratory in the hospital and at the bedside, at vast cost to ourselves.

In the attack to which we refer, not a word is said about the two years of time and labor (January, 1911—February, 1913) devoted by us, by our bacteriologists, by our Department of Experimental Medicine, and by hundreds of medical practitioners all over the United States to a searching investigation of every phase of the Phylacogens. Not a word is said about the hundreds of reputable physicians who have sent us between 4000 and 5000 reports of cases treated with Phylacogen; not a word about the thirty-four papers which have appeared in the medical press; not a word about the thirty-one meetings of medical societies before which papers have been read and the Phylacogens discussed; not a word about the one hundred thousand dollars which we have spent in an earnest, single-minded endeavor to ascertain and proclaim the truth; not a word about these forty-five pages of "Laboratory Data" which we sent out among our co-workers.

Our advertisements in the pharmaceutical journals are reproduced and made to represent Parke, Davis & Co. as caring not a malediction for the physician or the patient—as interested only in the shameful fruits of betraying the physician's confidence. Pray, study these advertisements and judge for yourself. It is perfectly true that we are advertising our Phylacogens extensively, that our advertising, supported by the remarkable merit of the Phylacogens, will create a market, and that the druggist should place the preparations in stock. What is there wicked in all this? Is this encouraging self-medication by the laity or counter-prescribing by the druggist? Have you ever heard of any druggist injecting a dose of Phylacogen?

Is there any possible question in your mind, doctor, that for the testing of new medicinal agents we have the most complete and the best laboratory facilities that can be found anywhere in America? As for clinical work, our Department of Experimental Medicine enjoys the support and co-operation of not less than 3200 of the best physicians in the country, with whom we are in constant correspondence and in almost daily contact through the twentythree representatives of this Department—physicians on whom we depend for the clinical proving of every new preparation before we place it on the market.

We close with a brief extract from a letter received on the 3d inst. from one of the most prominent medical editors in the United States:

"I have just read the mean attack on the Phylacogens in the current issue of the *Journal of the American Medical Association.* It fairly makes my blood boil, for I have had occasion to know a good deal about the great care your esteemed firm have taken to be sure of their position before offering a dollar's worth of these products to the market. If ever a firm pursued clean, ethical and scientific methods in developing a remedy for professional use, Parke, Davis & Co. have in the present instance."

Very truly yours,

PARKE, DAVIS & CO.,

FRANK G. RYAN, President.

The attack upon this great firm of *benefactors* by the *Octopus* is too contemptible for notice. Their reputation for all that is clean and ethical in their intercourse with the medical profession and their high character and standing in the commercial world puts them out of reach of the spiteful missiles,—mud slinging,—of that chronic sorehead, whose eyes are so bedimmed by envy and jealousy that they cannot see any good in anything that does not emanate from their own "Council of Pharmacy," or, seeing it, they are too mean to acknowledge it.—ED.

